# Evaluation of the HIV lay counselling and testing profession in South Africa

**DOI:** 10.1186/s12913-015-0940-y

**Published:** 2015-07-22

**Authors:** Aziza Mwisongo, Vuyelwa Mehlomakhulu, Neo Mohlabane, Karl Peltzer, Jacque Mthembu, Heidi Van Rooyen

**Affiliations:** School of Public Health, Faculty of Health Sciences, University of Witwatersrand, Johannesburg, South Africa; Wits Health Consortium-Clinical HIV Research Unit (CHRU), Department of Medicine, Faculty of Health Sciences, University of Witwatersrand, Johannesburg, South Africa; HIV/AIDS, STIs, and TB (HAST) Research Programme, Human Sciences Research Council (HSRC), Pretoria, Cape Town, Durban, Port Elizabeth South Africa; Department of Research & Innovation, University of Limpopo, Turfloop, South Africa; ASEAN Institute for Health Development, Madidol University, Salaya, Phutthamonthon, Nakhonpathom, 73170 Thailand

**Keywords:** Lay counsellors, HIV Counselling and Training (HCT), Training, HIV testing, Workspace

## Abstract

**Background:**

With the launch of the national HIV Counselling and Testing (HCT) campaign in South Africa (SA), lay HIV counsellors, who had been trained in blood withdrawal, have taken up the role of HIV testing. This study evaluated the experiences, training, motivation, support, supervision, and workload of HIV lay counsellors and testers in South Africa. The aim was to identify gaps in their resources, training, supervision, motivation, and workload related to HCT services. In addition it explored their experiences with providing HIV testing under the task shifting context.

**Methods:**

The study was conducted in eight of South Africa’s nine provinces. 32 lay counsellors were recruited from 67 HCT sites, and were interviewed using two questionnaires that included structured and semi-structured questions. One questionnaire focused on their role as HIV counsellors and the other on their role as HIV testers.

**Results:**

Ninety-seven percent of counsellors reported that they have received training in counselling and testing. Many rated their training as more than adequate or adequate, with 15.6 % rating it as not adequate. Respondents reported a lack of standardised counselling and testing training, and revealed gaps in counselling skills for specific groups such as discordant couples, homosexuals, older clients and children. They indicated health system barriers, including inadequate designated space for counselling, which compromises privacy and confidentiality. Lay counsellors carry the burden of counselling and testing nationally, and have other tasks such as administration and auxiliary duties due to staff shortages.

**Conclusions:**

This study demonstrates that HCT counselling and testing services in South Africa are mainly performed by lay counsellors and testers. They are challenged by inadequate work space, limited counselling skills for specific groups, a lack of standardised training policies and considerable administrative and auxiliary duties. To improve HCT services, there needs to be training needs with a standardised curriculum and refresher courses, for HIV counselling and testing, specifically for specific elderly clients, discordant couples, homosexuals and children. The Department of Health should formally integrate lay counsellors into the health care system with proper allocation of tasks under the task shifting policy.

## Background

Sub-Saharan Africa has the highest prevalence of Human Immunodeficiency Virus (HIV) infections globally. South Africa has the largest number of people living with HIV in the world [1], with an estimated 5.6 million people were living with the disease in 2011 [1]. HIV Counselling and Testing (HCT) has been said to be a key intervention in preventing new infections and reducing the impact of HIV/AIDS on individuals, families, communities, and society [2], and is an important gateway to receiving treatment, care and support [3].

The South African government launched the HCT campaign in April 2010, and by June 2011, over 13 million people had been tested for HIV [4]. Although there has been considerable success in making HCT accessible in South Africa, a shortage of trained HIV counsellors and trainers restricts the provision of HIV services, particularly in resource limited settings [5]. This is in contrast to the context of an increasing demand for HCT prompted by the rapid expansion of Antiretroviral (ART) services [6–8].

HCT services are provided mainly by trained HIV counsellors throughout sub-Saharan Africa [9]. In South Africa, the National Department of Health (NDOH) supports approximately 8000 lay counsellors with stipends to provide HIV counselling, and more recently, HIV testing [9]. The lay counsellor position is not part of the formal employment structure of the Department of Health, and under the supervision of their local facility manager, they provide HCT services on a part-time basis [10]. In 2010, the National HCT guidelines were changed to accommodate task shifting/sharing, and to allow non-health care workers to conduct HIV testing by means of a finger prick method. While the Department of Health trained new and previously trained lay counsellors in HIV testing in addition to counselling [2], its implementation has staggered, resulting in variations according to different institutions.

Several studies exploring the experiences of lay counsellors have cited the various structural challenges they face, including the lack of a designated counselling space, privacy, recognition and career structure [11], support and supervision by health care team members [12]. Others have cited a lack of training [11, 12] as well as low remuneration [13], all of which have been limited to the public setting. As HCT is now being provided in private as well as non-governmental settings which differ according to fees for services, with government facilities offering free services. Given this difference, it is important to study the experiences of lay counsellors in these institutions. This study therefore aimed to: explore the experiences of HIV counsellors and testers in both public and private settings; identify the gaps associated with providing counselling and testing; investigate the role of counsellors and testers under the task shifting context in public health facilities, and propose strategies for improving HCT services in South Africa.

## Methods

### Participants

A total of 73 HCT sites were approached to participate in the study, 67 agreed and six non-governmental organisations (NGO) refused, resulting in a response rate of 91.8 %. North West Province was excluded from the study due to difficulties obtaining study approval from the Provincial Department of Health. Of the 67 sites, with a total of 32 lay counsellors were interviewed by Human Science Research Council (HSRC) researchers, including the authors (AM, VM, NM and JM). There were no refusals from the counsellors who were approached to take part in the study.

### Sampling procedure

A two-stage sampling procedure was used, the first being to randomly select one district each from the eight provinces, Secondly, from a lists of public and private HCT sites, four public and three private facilities were conveniently sampled in each of the eight selected districts. In order to include different types of HCT models, public, private and NGOs were selected on the basis of the services provided e.g. service in a specific setting, using a specific model/approach, and/or providing services to a specific target population. Furthermore, effort was also made to include examples of Provider Initiated Counselling and Testing (PICT), community-based HCT with an outreach component, mobile and couples HCT, as part of a workplace-based health service that targeted specific high-risk populations (e.g. MSM, CSWs, drug users, youth), and provided with other services (e.g. TB diagnosis and treatment, family planning).

The study was approved by the Human Sciences Research Council Ethics Committee (Protocol REC 9/19/08/09; FWA number: 0000 6347 IRB number: 0000 3962) with approvals from Centres for Disease Control, NDOH, departments of health from the eight provinces (Eastern Cape, Free State, Gauteng, KwaZulu-Natal, Limpopo, Mpumalaga, Northern Cape, Western Cape). One counsellor/tester and/or one health worker who performed HIV tests were interviewed at each site, with the interviews taking place in a private setting. Written informed consent was obtained from all participants prior to the interviews, which were conducted in English or the local language, depending on the preference of the interviewee.

### Variables of interest

Lay counsellors were interviewed using two questionnaires that included semi-structured questions. One questionnaire focused on their role as HIV counsellors and the other on their role as testers. All questionnaires were pilot tested prior to the main study. The HIV counsellor questionnaire included items such as:

- *Biographics:* Background demographic data included ethnicity, age, education level, and employment status.
- *Work conditions/work load*: Four items regarding work conditions: “How many hours do you work a week?” and “How many clients do you see on average in a day”?
- *HCT services*: Three items to inquire about HCT services: 1. “On average how long do you spend on counselling each client?” 2. “Do any of your clients come back to you for follow-up counselling?” 3. “Have you had any experience with any of these types of HIV testing and counselling listed?” e.g. Couple’s counselling, Home-based counselling and testing etc.
- *Counselling process:* Seven items to assess counselling process, including: 1. “Have you had any problems with ensuring confidentiality?”2. “Have you have had any problems with ensuring privacy?”3. “Have you experienced any other difficulties in counselling and testing specific groups?”
- *HIV counselling training:* Seven items about training, including: 1. “Have you had any training in HIV counselling?” 2. “Who provided the training?” 3. “How long was the training?” 4. “Briefly, describe the training (content, quality and applicability).”
- *Support and supervision:* Four items to establish support and supervision provided, including: 1. “Do you have any formal form of supervision in your work as a counsellor?” and 2.“Are there any areas in which you would like more support or supervision?”
- *HIV testing:* Two items about HIV testing including: “Have you ever had an HIV test?” and “Do you know your HIV status?”

The HIV testing questionnaire included items that examined:

- *HIV testing training:* Two items about training on HIV testing: 1. “Were you provided with formal training for HIV testing?” 2. “If YES, mention the name of the training organisation.”
- *Tester’s work conditions/work load:* Four items to measure work conditions, including: “How many hours do you work a week?”, “How many clients on average do you test in a day?” and “What other job tasks and responsibilities do you have besides HIV testing?”
- *HIV testing experience:* One question about counsellors’ testing experience: “How long have you been doing HIV testing?”

### Data Analysis

All quantitative data was analysed using the IBM Statistical Package for Social Scientists (SPSS, Version 20.0). Cross-tabulations and descriptive statistical analysis were undertaken. Content analysis was used to analyse open-ended questions. Initial categories for the analysis were drawn from the interview guides and themes and patterns emerged after reviewing the data within and across respondent groups [14]. Transcripts were reviewed by co-investigators and a preliminary list of codes was developed and subsequently refined [15]. Data were coded and reviewed, major trends and cross-cutting themes were identified and issues for further exploration were prioritised for final analysis. Coding discrepancies were resolved through co-investigator discussion and consensus.

## Results

### Sample characteristics

Of the 32 lay councillors, 27 (84.3 %) were females and 5 (15.6 %) were males, and the mean age was 35 years (SD = 8.6), with the oldest being 53 and the youngest being 19. More than half (17, 53 %) had completed grade 12 (12 years of education from primary level) and (15, 47 %) have an education lower than grade 12, while (17, 53.1 %) work in rural areas, (921.1 %) in peri-urban, 5(16 %), in urban settings and 1(3.1 %) in an informal settlement area. A total of 7 (21.8 %) lay counsellors worked in hospitals, 19 (59.4 %) in primary health care clinics, 3 (9.4 %) in stand-alone HCT sites, 2 (6.3 %) in mobile HCT service, and 1(3 %) in community centres (see Table 1).

**Table 1:** Socio-demographic and work place characteristics of lay counsellors

Variable (N=32)	n (%)
Sex	
Males	5 (15.6)
Females	27 (84.4)
Age (years)	
19-24	3 (9.4)
25-35	16 (50.0)
36-40	3 (9.4)
40>	
Geographical type of the lay counsellor’s work-place	
Urban area	5 (15.6)
Peri urban area	9 (28.1)
Informal settlement	1 (3.1)
Rural area	17 (53.1)
HIV Counselling and Testing type of the lay counsellor’s workplace	
Hospitals	7 (21.8)
Health care clinics	19 (59.4)
Standalone HCT sites	3 (9.4)
Mobile HCT service	2 (6.3)
Community centres	1 (3.1)

### Training in HIV counselling and HIV testing

Most lay counsellors (31, 97 %) reported that they have received HIV counselling training, which was received from different organisations, such as the Department of Health, educational institutions, NGOs and as part of a research study. Regarding the number of days for counselling training, 8 (25 %) attended for less than 10 days, 15 (46.9 %) for 10 days, and 9 (28.1 %) for more than 10 days. A total of 15 (46.9 %) lay counsellors felt that they have received more than adequate training in HIV counselling, while 11 (37.5 %) felt that the training had been adequate and 6 (18.8 %) felt it was inadequate. Almost all the lay counsellors 30 (93.8 %) indicated that they would like to have additional training, with 2 (6 %) feeling that it was not necessary. Some of the areas they reported in need of additional training were updates on ARVs, Prevention of Mother to Child Transmission of HIV/AIDS (PMTCT), counselling pregnant teenagers and same sex partners, and TB and HIV integration. When asked if they knew about the new HCT policy, 15(46.9 %) affirmed this, while 17(53.1 %) said they know about it. Of those who were aware of it, only 8 (25 %) had incorporated the guidelines into their work, while 24 (75 %) had not done so.

Almost all the lay counsellors (31, 96.8 %) had received formal training in HIV testing, with 1(3.1 %) person not responding to the question. Four of the six counsellors who had not received formal training in HIV testing were from NGOs. A range of organisations were mentioned as providing HIV testing training, including NGOs, universities, and Departments of Health at national, provincial and district levels.

### Support and supervision

A total of 37 (86.1 %) of the lay counsellors reported having some kind of formal counselling supervision at work, while 5 (11.6 %) reported the opposite. More than half (17, 53.1 %) reported that there were no areas in which they would like more support or supervision, while 15 (46.9 %) indicated the contrary. For those who needed support, this related to issues of inspiration, emotional support and debriefing. In general, they felt that they were undermined by other health professionals because of their lack of health related education, as stated by one below.

*“I feel our work in this facility gets undermined by other professionals probably because of the level of our education.” [23 yr old female counsellor, Government health service]*

### HIV counselling process and its challenges

Challenges with the lack of privacy and confidentiality during counselling sessions were reported by 15 (46.9 %) and 4 (12.5 %) respectively, these being more apparent in the government health services than the private and NGO sectors. The main limitation with ensuring privacy was the lack of dedicated space/rooms for HCT, as expressed by one of the counsellors:

*“Yes, because there are no private HCT rooms, so sometimes we use other people’s offices.” [32 yr old male counsellor, government health service]*

In some health facilities, HCT duties were shared among all staff members who were not counsellors, rather than being allocated to permanent designated officers. Some of the counsellors felt that this contributed to difficulties in executing their tasks and maintaining a consistent level of confidentiality, as no one was held liable for the delivery of HCT services, as stated below:

*“Previously, when nurses did counselling through rotation, it was difficult to ensure and maintain confidentiality.” [49 yrs. old female counsellor, government health service]*

Similarly, patients attending the health facilities were blamed for spreading news on other patients HIV status, as reported below:

“*Usually it is the patients who attend the hospital that spread the news of people’s status, which is usually blamed as counsellor’s poor maintenance of confidentiality.” [25 yr. old male counsellor, government health service]*

Difficulties during counselling session were expressed by 26 (81.3 %) of the counsellors. The commonest problem was the lack of counselling skills when faced with difficult situations, such as counselling an individual with a positive result, handling discordant couples, counselling clients of extreme old and young age groups, counselling gay/lesbian clients and sex workers. Table 2 presents some statements regarding the situations and difficulties counsellors faced.

**Table 2:** Quotes of counsellors on the challenges faced with some counselling sessions and their implication

Quotes of lay counsellors	Implications
*“If a patient does not accept his status (HIV positive) then I have to deal with the emotions as well.” [26 yr old female counsellor, NGO]*	Providing emotional support
*“When a couple is discordant and one of them already knows his/her status, it usually leads to a fight.” [38 yr old female counsellor, NGO]*	Mediating a couples fight
*“When a 12 to 13 year old is found to be positive, they cry and you are compelled to hug them as a parent.” [50 yr old female counsellor, government health service]*	Providing emotional support
*“Sometimes I have to counsel an older client e.g. above 60 yrs because they are in denial and not easy for them to understand why they are positive.” [49 yr old female counsellor, government health service]*	Dealing with denial

### Challenges with HIV testing

The majority of the lay counsellors (24, 75 %) had testing experience of between a few months and five years. All 32 counsellors declared that they perform an HIV screening test at the HCT sites, and the majority did confirmation tests at the same site. Counsellors also indicated specific challenges in relation to HIV testing, such as: health systems issues, testing guidelines and sensitivity of test kits, low incentives/lack of recognition/high workloads, and stigma/fear/denial of HIV testing, as illustrated in Table 3.

**Table 3:** HIV testing issues

Category of issues	Specific issues
Health systems issues	- Frequent stock outs of test kits affecting clients participation
	- Lack of working space for counsellors and testers
	- Lack of some important work materials e.g. stopwatches
Testing guidelines and sensitivity of test kits	- Low sensitivity of the G-ocean brand
	- High usage of ELISA due to mistrust of rapid tests
	- Constant changes in test kits
	- Current test kits are not easy to recognise if there is little buffer or blood
	- Often different results between screening and confirmatory tests
Low incentives/ lack of recognition/ workload	- Low salary compared to tasks
	- Delays in salaries
	- Not considered as part of health facility staff
	- Lack of re-training for counsellors and testers
	- Extra work in the facility due to HCT but limited staff
	- HCT too demanding and stressful, need more staff
	- Emotional stress of counsellors and testers , thus need counselling and psychological support themselves
Stigma/fear/denial of HIV testing	- Fear of staff members to test in the facility where they work
	- Difficulties of testing oneself
	- Difficulties of testing couples especially in relational issues
	- Many men in denial of their results
	- Positive youths react negatively after receiving results
	- Difficult to maintain confidentiality in small communities

### Counsellors work conditions/workload

The lay counsellors worked an average of 33 hours per week, with a minimum of seven and a maximum of 70 hours. An average of 20 hours was spent on HIV counselling and testing per week, with a minimum of two and a maximum of 40 hours. They reported counselling an average of 12 clients per day, testing from nine to 25 clients on a busy day. Many of the lay counsellors also performed other duties apart from HIV counselling such as HIV testing, ART adherence counselling, PMTCT services, coordinating support groups, providing home based and clinical care, and pre-packing patient medication. Other duties included assisting with administration, causality care, theatrical duties, reception, filling in lab forms, providing family planning services, health education and promotion, as well as Orphan and Vulnerable Children (OVC) care, conducting polymerase chain reaction (PCR) testing and pap smears, sputum collection, pill count, clean hazard boxes, and training and mentoring e.g. of auxiliaries.

## Discussion

The national HCT policy guidelines stipulate that all service providers should be trained according to the National Minimum Standards for Counselling and Testing [2]. In this study, the majority of lay counsellors reported that they received adequate training for both HIV is counselling and testing. Similar studies in this area indicated that many counsellors were found to be untrained [11, 16]. Studies have indicated that training varies in length and content, depending on the training organisation [17], as was the case in this study, as they received training from different HCT organisations. Although not ascertained in this study, there could also be variation of training among lay counsellors in the same health facility. All of these could partly explain the lack of confidence by some counsellors in counselling certain groups/situations.

It has been argued that counsellor support and supervision is important, as it helps to reduce stress, and thereby strengthens the quality of counselling procedures [18]. The majority of the lay counsellors in this study seemed comfortable with the amount of supervision they received, this being contrary to other studies, where they reported little or no supervision [11, 19]. However, our study reiterates the fact found by Malema et al. [20] in Limpopo Province, where counsellors lamented the lack of emotional support from their superiors. Furthermore, our study also reveals a sense of low recognition among fellow health workers thought to be compounded by lack of permanent positions and low remuneration. These findings echo results from other studies, where lay counsellors also expressed similar discontent with their working conditions [19].

The emotional dissatisfaction was further exacerbated by a poor work environment, such as inadequate infrastructure, which impedes privacy and confidentiality practices during counselling sessions, this being a major concern among the counsellors. This seems to be an on-going problem in South Africa, as also expressed by a study of lay counsellors in the Eastern Cape in a study by Peltzer and Davids in 2011 [11]. Similarly, in a study conducted in Cape Town, counsellors also reported that they lacked designated counselling space and privacy, as they shared space with other health care workers in the health facility [21]. Haffejee [22] noted that as a result of inadequate facilities and high patient loads, counselling services were usually rushed to accommodate other services. This is a health system concern, as the lack of privacy comprises confidentiality and contribute to making counselling sessions less effective [22], which could also deter clients from seeking care.

The results also indicate that lay counsellors work an average of 34 hours per week, and perform other duties such as administration and auxiliary services. The World Health Organisation, in their task shifting policy, recommended several tasks deemed essential for preventing HIV transmission that can be allocated to community health workers, of which administration forms part [23]. This is a concern, as the increasing demand for HCT and Anti-retroviral treatment (ART) services results in large numbers of patients need to be attended to by counsellors. HIV counselling and testing is a strenuous job on its own, and to add on other duties may lead to consistent counsellor burn-out. It would be advisable that lay counsellor’s duties be streamlined and structured in accordance with the services provision standards.

This study did not aim to investigate to what extent has task shifting has been implemented. However task shifting was reported as a common context under which HIV counsellors and testers operated. In line with this, most had received inclusion of testing as part of the task shifting process, with which they were satisfied. Challenges were reported around frequent test kit shortages, which affect clients’ participation. Some counsellors also lamented their high workload, as they had to perform other tasks related to administration and auxiliary duties. The task-shifting policy of allowing lay counsellors to do HIV testing has also been implemented in countries such as Zambia [24]. However, this study suggests that better coordination is needed to ensure a balance between counselling and testing tasks and other duties, in public health facilities in South Africa in particular.

## Study limitations

Caution should be taken when interpreting these results due to the study limitations. The study sample not representative of all lay counsellors in South Africa, and the findings cannot be generalized to the experiences of these service providers elsewhere in the country. The study sample had few private facilities compared to public ones, making it difficult to carry out comparative analysis. The training assessment had limitations as the researchers did not make reference to the counsellor’s minimum standard in determining if they were properly trained. Lastly, as the study was not intended to assess task shifting, it is unwise to make any in-depth conclusions about its execution in relation to lay counsellors.

## Conclusion

The results indicate that lay counsellors and testers play an important role in HCT provision in South Africa. However, their role is challenged by a lack of standardised counselling and testing training, designated working areas to maintain privacy and confidentiality, and specific counselling skills for marginal groups?. While lay counsellors have been absorbed into the health care system under the task shifting policy, there is evidence that they are overburdened with HCT work as well as other administration and auxiliary duties due to staff shortages. There is also evidence of low morale and motivation, partly affected by poor remuneration and recognition by fellow permanently positioned health workers. It is essential for counsellors to be provided with adequate work spaces that allow for privacy and confidentiality. The government of South Africa should consider formally integrating lay counsellors into the health care system with clearly identified tasks under the task shifting policy. A standardised curriculum for HIV counselling and testing training should be adapted to ensure quality HCT services are provided to clients. Refresher training is recommended to make sure counsellors are always well aware of what is expected of them. Lastly, the development of counsellor training curriculum to enable them to cope with certain aspects of the HIV/AIDS counselling process (discordant couples, elderly, children and homosexual clients) should be considered.
